# Peripheral blood T-cell subsets combined with EGRIS score predict the need for mechanical ventilation in Guillain–Barré syndrome

**DOI:** 10.3389/fimmu.2026.1747416

**Published:** 2026-02-04

**Authors:** Feihong Jia, Xinrui Wang, Yuan Chen, Yunshu Li, Xinshu Du, Hongping Chen, Di Zhong

**Affiliations:** Department of Neurology, The First Affiliated Hospital of Harbin Medical University, Harbin, China

**Keywords:** CD4+ T cell, CD8+ T cell, Guillain–Barré syndrome, mechanical ventilation, peripheral blood lymphocyte subset

## Abstract

**Background:**

The pathogenesis of Guillain–Barré syndrome (GBS) may involve lymphocyte-mediated immune mechanisms. This study aimed to investigate the relationship between peripheral blood lymphocyte subsets and disease onset, severity, and the need for mechanical ventilation in patients with GBS.

**Methods:**

This cohort study included 55 patients with GBS and 58 healthy controls. The association between peripheral blood lymphocyte subsets and the onset and severity of GBS was investigated. Among the GBS patients, 26 were classified into a non-mechanical ventilation group and 29 into a mechanical ventilation group, and the predictive value of peripheral blood lymphocyte subsets for mechanical ventilation was evaluated. Furthermore, the study examined the expression of relevant lymphocyte subsets in the sciatic nerve of experimental autoimmune neuritis (EAN) rat models at different disease stages.

**Results:**

ROC curve analysis showed that both CD8^+^ T cell counts and NK cell counts had moderate discriminative ability in distinguishing patients with GBS from controls. The AUC for CD8^+^ T cell counts was 0.73 (95% CI: 0.637–0.836), with an optimal cutoff value of 394.39 cells/μL, while the AUC for NK cell counts was 0.81 (95% CI: 0.731–0.893), with an optimal cutoff value of 147.7 cells/μL. Total lymphocyte counts, total T cell counts, CD8^+^ T cell counts, and CD4^+^ T cell counts were negatively correlated with Hughes peak score (all *P* ≤ 0.005). Logistic regression showed that higher peripheral CD4^+^ T cell counts was associated with reduced need for mechanical ventilation (OR = 0.997; 95% CI: 0.994–1.000, *P* = 0.032). The combined ROC analysis of CD8^+^ T cell counts, CD4^+^ T cell counts, and the EGRIS score demonstrated good discriminative ability for identifying GBS patients who required mechanical ventilation, with an AUC of 0.87 (95% CI, 0.772–0.971, *P* < 0.001). In the EAN model, CD4^+^ T cell expression was increased in sciatic nerve tissue.

**Conclusion:**

Peripheral blood lymphocyte subsets show potential value in differentiating disease severity and the need for mechanical ventilation in patients with GBS, highlighting the clinical significance of immune cell profiling in risk stratification.

## 1 Introduction

Guillain–Barré syndrome (GBS) is an acute immune-mediated disorder of the peripheral nervous system characterized by rapidly progressive weakness and areflexia. Increasing evidence indicates that a variety of bacterial and viral infections, including cytomegalovirus, Zika virus, Epstein–Barr virus, Mycoplasma pneumoniae, and SARS-CoV-2 (COVID-19), are implicated in the pathogenesis of GBS (1–3). These infections are thought to trigger aberrant immune responses through mechanisms such as molecular mimicry, in which antibodies produced against pathogen antigens cross-react with components of peripheral nerves, leading to inflammation, demyelination, and axonal injury.

In the experimental autoimmune neuritis (EAN) rat model of GBS, circulating lymphocytes and macrophages have been observed to infiltrate and adhere to the endoneurial interstitium of the sciatic nerve (4). Exogenous administration of non-specifically activated T cells induces perivascular infiltration within the endoneurium of peripheral nerves, which, in combination with anti-myelin antibodies, leads to dose-dependent demyelination and axonal degeneration (5).

Recent studies have shown that patients with GBS harbor self-reactive memory CD4^+^ T cells with a Th1-like phenotype, as well as CD8^+^ T cells that recognize peripheral nerve myelin antigens. Moreover, myelin-reactive T cells have been identified within the peripheral nerves of one patient, further supporting the notion that T cells play a pivotal role in mediating peripheral nerve pathology in GBS (6).

In this study, clinical data and laboratory findings from patients with GBS were collected to investigate the associations between peripheral blood lymphocyte subsets—particularly CD4^+^ T cells and CD8^+^ T cells—and disease onset, severity, and the requirement for mechanical ventilation.

## 2 Methods

### 2.1 Clinical data collection

This single-center, retrospective cohort study enrolled 55 patients with acute GBS. All patients were hospitalized at the first affiliated hospital of Harbin medical university between January 2018 and October 2024 and underwent peripheral blood lymphocyte subset testing. Eligible patients were aged ≥18 years and had complete clinical and laboratory data. Patients with incomplete records or peripheral neuropathy due to other causes (e.g., periodic paralysis, paraneoplastic neuropathy, Sjögren’s syndrome, drug-induced neuropathy, acute exacerbation of chronic inflammatory demyelinating polyneuropathy, or heavy metal poisoning) were excluded. A total of 58 healthy adults who underwent the same testing at the hospital’s physical examination center during the same period served as controls.

Demographic and laboratory data were collected for all participants. For healthy controls, age, sex, and peripheral blood lymphocyte subsets were recorded. For GBS patients, clinical data included age, sex, onset season, prodromal history (diarrhea, upper respiratory tract infection, vaccination, COVID-19, or other events), clinical and electrophysiological classification, and functional scores, including the Medical Research Council (MRC) score, Hughes score (7), and Erasmus GBS Respiratory Insufficiency Score (EGRIS) score (8). Laboratory data comprised complete blood counts, lipid profile, and peripheral blood lymphocyte subset measurements. Peripheral blood samples from GBS patients were collected upon admission and before the start of treatment. All blood tests were performed by professional laboratory personnel in the Department of Immunology, First Affiliated Hospital of Harbin Medical University. This study retrospectively collected data from standardized clinical laboratory reports. Analysis of lymphocyte subsets was performed using flow cytometry, following standard clinical laboratory procedures.

### 2.2 Animal models

A 200 μL immune complex emulsion was prepared by combining 230 μg peripheral nerve myelin P2 peptide (amino acids 53–78; TESPFKNTEISFKLGQEFEETTADNR), 2 mg H37RA Mycobacterium tuberculosis, 100 μL incomplete Freund’s adjuvant, and 100 μL normal saline. The emulsion was vortexed on ice and injected subcutaneously approximately 1 cm from the base of the tail in Lewis rats. Following immunization, neurological function was assessed daily in the rats using the 0–10 scoring system described by Enders et al (9).

### 2.3 Immunofluorescence

Rats were anesthetized with 0.3% sodium pentobarbital (65 mg/kg, intraperitoneal injection). Cardiac blood was collected, and bilateral sciatic nerves were exposed by blunt dissection, removed, and embedded in Tissue-Tek OCT compound. Tissue blocks were cut into 10-μm-thick sections using a freezing microtome, fixed with 4% paraformaldehyde for 15 minutes, rinsed with PBS, and blocked with Triton X-100 buffer for 1 hour at room temperature. Sections were incubated with mouse anti-CD4 monoclonal antibody (Protein tech) overnight at 4°C and then with FITC-conjugated goat anti-mouse IgG (H + L) (Boster Biological Technology) for 1 hour at room temperature. After washing with PBS, sections were mounted with antifade mounting medium containing DAPI, and images were acquired using a Nikon upright fluorescence microscope.

### 2.4 Statistical analysis

Clinical data were analyzed using SPSS 25.0, and animal data were analyzed and plotted using GraphPad Prism 9.5. The normality of continuous variables was assessed using the Shapiro-Wilk test. Variables that were normally distributed are presented as mean ± standard deviation and compared between groups using independent samples t-tests, whereas non-normally distributed variables are presented as median and interquartile range (IQR) and compared using Mann-Whitney U tests. One-way ANOVA followed by Tukey’s *post hoc* test was used for comparisons among multiple groups. Categorical variables were expressed as n (%) and compared using the chi-square test. *P* value < 0.05 was considered statistically significant.

## 3 Results

### 3.1 Association of peripheral blood lymphocyte subsets with the onset and severity of GBS

Statistical analyses were conducted on peripheral blood lymphocyte subsets from 58 healthy controls and 55 patients with GBS. The proportion of females was higher among healthy controls (23, 39.7%), while males were predominant in the GBS group (32, 58.2%) (*P* = 0.049). There was no significant difference in age between the two groups (**Supplementary Table 1**).

**Figure 1:**
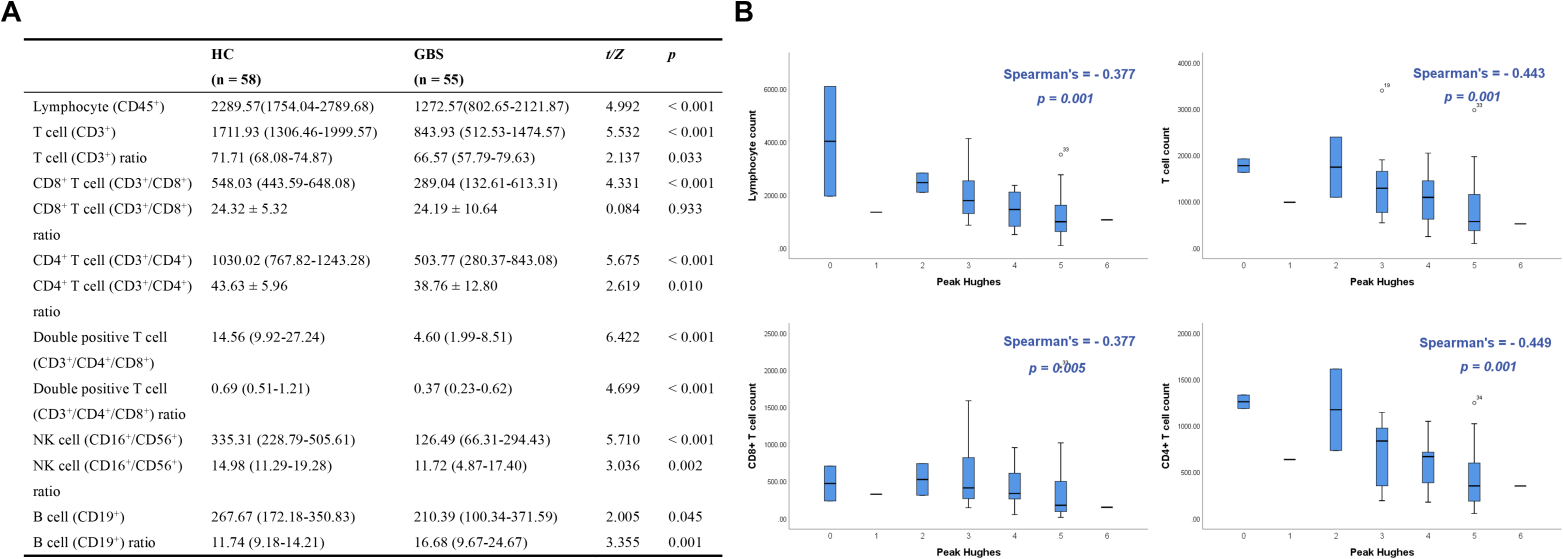
Peripheral blood lymphocyte subsets in Guillain-Barré syndrome (GBS) patients and healthy controls. **(A)** Peripheral blood lymphocyte subset of healthy controls and GBS patients were expressed as mean ± standard deviation or median (interquartile range), and comparisons between the two groups were performed using the t-test or Mann–Whitney U test. *P* value < 0.05 was considered statistically significant. **(B)** Spearman correlation analysis of peripheral blood lymphocyte subsets in healthy controls and GBS patients. *P* < 0.05 was considered significant.

**Figure 2:**
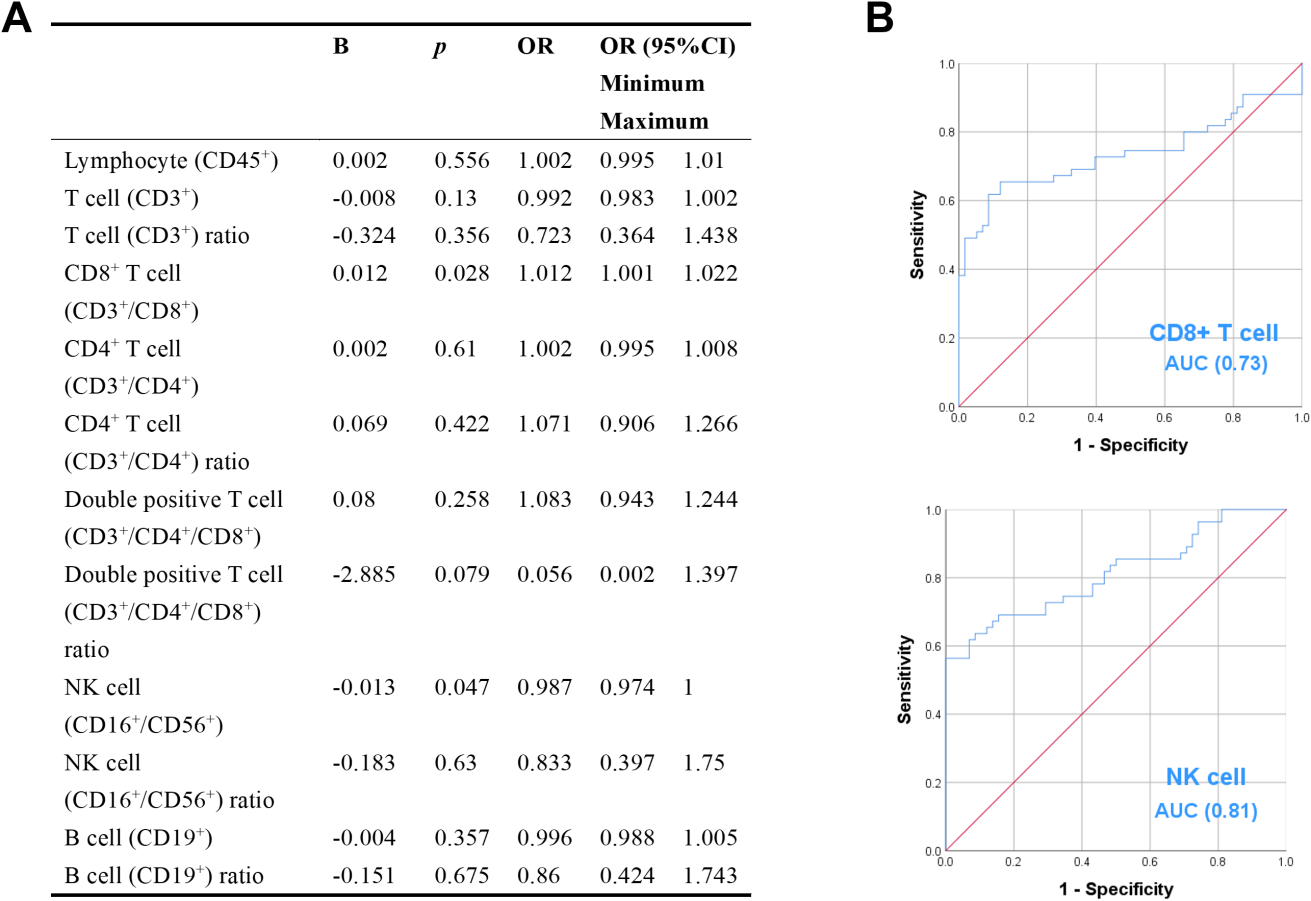
The association between peripheral blood lymphocyte subsets and Guillain-Barré syndrome (GBS). **(A)** Multivariate logistic regression analysis of peripheral blood lymphocyte subsets in patients with GBS. *P* < 0.05 was considered significant. **(B)** ROC curves of CD8^+^ T and NK cell counts for predicting the onset of GBS. *P* < 0.05 was considered significant.

Except for the proportion of CD8^+^ T cells, the levels of peripheral blood lymphocyte subsets in patients with GBS were significantly lower than those in healthy controls (**Figure 1A**). Logistic regression analysis showed that an increased CD8^+^ T cell counts was associated with a higher likelihood of GBS (B = 0.012, OR = 1.012, 95% CI: 1.001–1.022, *P* = 0.028), whereas an increased NK cell counts was associated with a lower likelihood of GBS (B = −0.013, OR = 0.987, 95% CI: 0.974–1.000, *P* = 0.047) (**Figure 2A**). In this retrospective analysis, ROC curve analysis showed that both CD8^+^ T cell counts (AUC = 0.73, 95% CI: 0.637–0.836, *P* < 0.001; optimal cutoff: 394.39 cells/μL) and NK cell counts (AUC = 0.81, 95% CI: 0.731–0.893, *P* < 0.001; optimal cutoff: 147.7 cells/μL) had discriminative ability for identifying patients with GBS. Specifically, higher CD8^+^ T cell counts were associated with a higher likelihood of GBS, whereas higher NK cell counts were associated with a lower likelihood of GBS. However, their prospective predictive value warrants further validation in future studies (**Figure 2B**).

Spearman correlation analysis between peripheral blood lymphocyte subsets and peak Hughes scores in patients with GBS revealed significant negative correlations between peak Hughes scores and the counts of total lymphocytes, T cells, CD8^+^ T cells, and CD4^+^ T cells (**Figure 1B**).

### 3.2 Clinical features of GBS patients with and without mechanical ventilation

The above findings indicate that peripheral blood lymphocyte subsets are associated with the pathogenesis and severity of GBS. Since severe GBS patients often require mechanical ventilation, we further investigated the association between peripheral blood lymphocyte subsets and the need for mechanical ventilation in GBS patients.

GBS patients requiring mechanical ventilation (59.38 ± 14.73 years) were significantly older than those not requiring mechanical ventilation (49.65 ± 14.99 years) (*P* = 0.019) (**Table 1**). There were no statistically significant differences between the two groups in terms of gender, season of onset, prior medical history, or the presence of diabetes. Mechanically ventilated GBS patients had longer hospital stays, a higher incidence of cranial nerve palsy and autonomic dysfunction, more frequent dyspnea, and higher EGRIS scores. In contrast, non-ventilated GBS patients exhibited longer onset-to-admission intervals and a higher frequency of sensory disturbances (**Tables 1**, **2**). The acute inflammatory demyelinating polyneuropathy (AIDP) subtype was the most common clinical and electrophysiological classification in both ventilated and non-ventilated GBS patients; however, neurological function was markedly worse in patients requiring mechanical ventilation than in those who did not (**Table 2**).

**Table 1:** Clinical characteristics of patients with GBS.

Clinical Characteristics	No mechanical ventilation (n = 26)	Mechanical ventilation (n = 29)	*χ2/t/Z*	*p*
Age (years)	49.65 ± 14.99	59.38 ± 14.73	2.425	0.019
Gender			1.357	0.244
Male	13 (50.0)	19 (65.52)		
Female	13 (50.0)	10 (34.5)		
Season of disease			4.419	0.220
Spring	4 (15.4)	3 (10.3)		
Summer	10 (38.5)	9 (31.0)		
Autumn	7 (26.9)	4 (13.8)		
Winter	5 (19.2)	13 (44.83)		
Precursor history			3.066	0.547
None	14 (53.8)	12 (41.4)		
Diarrhea	3 (11.5)	6 (20.7)		
Respiratory tract infection	8 (30.8)	7 (24.1)		
COVID-19	0 (0.00)	1 (3.4)		
After surgery	1 (3.8)	3 (10.3)		
Number of days in hospital	8.5 (6.75-10.25)	14.00 (7.00-26.50)	2.403	0.016
EGRIS	2.00 (1.00-3.25)	5.00 (3.00-6.00)	3.874	< 0.001
Diabetes			0	1.000
No	21 (80.8)	24 (82.8)		
Have	5 (19.2)	5 (17.2)		
Sensory impairment			9.814	0.002
No	6 (23.1)	20 (69.0)		
Have	20 (76.9)	9 (31.0)		
Cranial nerve palsy			6.623	0.010
No	18 (69.2)	10 (34.5)		
Have	8 (30.8)	19 (65.5)		
Autonomic dysfunction			13.173	< 0.001
No	19 (73.1)	7(24.1)		
Have	7(26.9)	22(75.9)		
trouble breathing			27.644	< 0.001
No	21 (80.8)	3 (10.3)		
Have	5 (19.2)	26 (89.7)		

Data are presented as mean ± standard deviation, median [interquartile range], or number (frequency percentage).

EGRIS, Erasmus GBS Respiratory Insufficiency Score; COVID-19, coronavirus disease 2019.

**Table 2:** Neurological function scores and clinical/electrophysiological classification in patients with GBS.

Scoring and Classification	No mechanical ventilation (n = 26)	Mechanical ventilation (n = 29)	*χ2/Z*	*p*	Total GBS patients (n = 55)
Onset-to-admission (days)	5.50 (3.00-10.00)	3.00 (1.5-5.00)	2.213	0.027	4.00 (2.00-7.00)
Admission MRC	54.00 (40.50-58.00)	24.00 (4.00-46.00)	3.286	0.001	42.00 (42.00-54.00)
Peak MRC	49.00 (36.00-58.00)	0.00 (0.00-16.00)	5.697	< 0.001	22.00 (0.00-50.00)
Discharge MRC	54.00 (46.50-58.00)	12.00 (0.00-27.00)	5.430	< 0.001	30.00 (6.00-54.00)
Admission Hughes	3.00 (2.00-4.00)	4.00 (3.50-4.00)	2.390	0.017	4.00 (3.00-4.00)
Peak Hughes	4.00 (3.00-4.00)	5.00 (5.00-5.00)	6.319	< 0.001	5.00 (4.00-5.00)
Discharge Hughes	3.00 (2.75-4.00)	5.00 (4.00-5.00)	5.070	< 0.001	4.00 (3.00-5.00)
Clinical classification			0.438	0.803	
AIDP	18 (69.2)	22 (75.9)			40 (72.7)
AMAN	5 (19.2)	5 (17.2)			10 (18.2)
AMSAN	3 (11.5)	2 (6.90)			5 (9.1)
MFS	0 (0.00)	0 (0.00)			0 (0.00)
ASN	0 (0.00)	0 (0.00)			0 (0.00)
Electrophysiological classification			2.168	0.538	
Demyelinating	14 (53.8)	20 (69.0)			34 (61.8)
Axonal injury	8 (30.8)	7 (24.1)			15 (27.3)
F wave abnormality	3 (12.00)	2 (6.9)			5 (9.26)
No abnormality	1 (3.8)	0 (0.00)			1 (1.8)

Data are presented as mean ± standard deviation, median [interquartile range], or number (frequency percentage).

GBS, Guillain–Barré Syndrome; MRC, Medical Research Council; AIDP, acute inflammatory demyelinating polyneuropathy; AMAN, Acute motor axonal neuropathy; AMSAN, acute motor and sensory axonal neuropathy; MFS, miller fisher syndrome; ASN, acute sensory neuropathy.

Compared with GBS patients who did not receive mechanical ventilation, those who required mechanical ventilation had lower lymphocyte counts, significantly higher inflammatory markers (including the neutrophil-to-lymphocyte ratio (NLR) and platelet-to-lymphocyte ratio (PLR)), and lower total cholesterol levels (**Supplementary Table 2**).

### 3.3 Predictive role of peripheral blood lymphocyte subsets and EGRIS in mechanical ventilation of GBS patients

Intergroup comparison of peripheral blood lymphocyte subsets between mechanically ventilated and non-ventilated GBS patients showed that lymphocyte (*P* = 0.008), T cell (*P* = 0.007), CD8^+^ T cell (*P* = 0.007), and CD4^+^ T cell (*P* = 0.008) counts were significantly lower—approximately half— in the mechanically ventilated group compared with the non-ventilated group (**Table 3**). The EGRIS score is a clinical tool used to predict the risk of respiratory failure and the need for mechanical ventilation in patients with GBS. In this study, the median EGRIS score among mechanically ventilated GBS patients was 5, indicating a high risk of requiring ventilatory support (**Table 1**).

**Table 3:** Peripheral blood lymphocyte subsets in patients with GBS.

Lymphocyte Subsets	No mechanical ventilation (n = 26)	Mechanical ventilation (n = 29)	*t/Z*	*p*
Lymphocyte (CD45^+^)	1812.03 (1130.48-2308.11)	1041.99 (660.90-1626.42)	2.664	0.008
T cell (CD3^+^)	1219.78 (758.47-1544.46)	609.16 (380.03-1160.86)	2.714	0.007
T cell (CD3^+^) ratio	68.45 ± 14.44	64.49 ± 14.84	1.079	0.281
CD8^+^ T cell (CD3^+^/CD8^+^)	357.44 (259.63-625.22)	171.26 (97.25-502.88)	2.697	0.007
CD8^+^ T cell (CD3^+^/CD8^+^) ratio	25.20 ± 9.19	23.28 ± 11.88	0.664	0.509
CD4^+^ T cell (CD3^+^/CD4^+^)	714.93 (370.24-994.07)	356.70 (189.54-600.79)	2.647	0.008
CD4^+^ T cell (CD3^+^/CD4^+^) ratio	40.01 ± 13.18	37.63 ± 12.56	0.684	0.497
Double positive T cell (CD3^+^/CD4^+^/CD8^+^)	5.58 (2.17-10.73)	3.98 (1.53-7.98)	1.264	0.206
Double positive T cell (CD3^+^/CD4^+^/CD8^+^) ratio	0.35 (0.25-0.70)	0.38 (0.25-0.70)	0.826	0.409
NK cell (CD16^+^/CD56^+^)	142.52 (83.90-313.26)	108.51 (42.37-262.31)	1.416	0.157
NK cell (CD16^+^/CD56^+^) ratio	10.43 (4.54-17.12)	12.14 (5.24-18.48)	0.287	0.774
B cell (CD19^+^)	259.79 (130.52-389.00)	186.15 (78.76-247.48)	1.382	0.167
B cell (CD19^+^) ratio	13.70 (9.54-23.32)	19.10 (10.37-25.66)	1.037	0.300

Data are presented as mean ± standard deviation, median [interquartile range]. Cell counts (cells/μL). Cell ratio (%).

**Figure 3:**
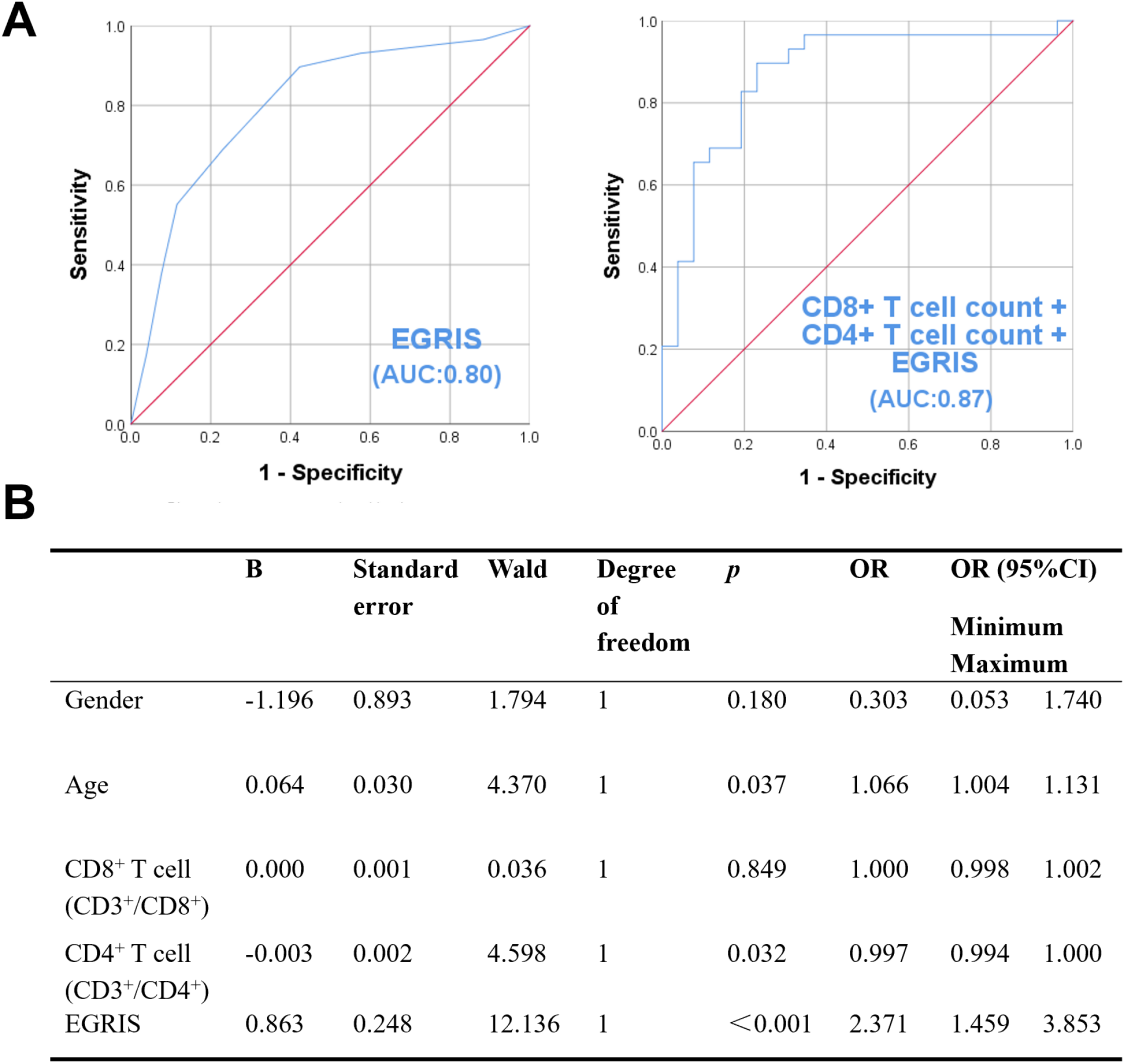
Association of peripheral blood lymphocyte counts with the need for mechanical ventilation in patients with Guillain–Barré syndrome (GBS). **(A)** ROC analysis of CD8^+^ T cell and CD4^+^ T cell counts and the predictive value of Erasmus GBS Respiratory Insufficiency Score (EGRIS) for mechanical ventilation in GBS. *P* < 0.05 was considered significant. **(B)** Multivariate logistic regression analysis of gender, age, CD8^+^ T cell counts and CD4^+^ T cell counts and EGRIS in GBS patients requiring mechanical ventilation. *P* < 0.05 was considered significant.

To assess the independence of predictive factors, lymphocytes subsets and T cells were excluded, and a multivariate logistic regression analysis was performed including sex, age, CD8^+^ T cell counts, CD4^+^ T cell counts, and EGRIS score to identify independent risk factors for mechanical ventilation in GBS patients. The analysis showed that increased age and higher EGRIS score were independent risk factors for mechanical ventilation (age: B = 0.064, OR = 1.066, 95% CI: 1.004–1.131, *P* = 0.037; EGRIS: B = 0.863, OR = 2.371, 95% CI: 1.459–3.853, *P* < 0.001), whereas a higher CD4^+^ T cell counts was associated with a reduced risk of mechanical ventilation (B = -0.003, OR = 0.997, 95% CI: 0.994–1.000, *P* = 0.032) (**Figure 3B**).

ROC analysis demonstrated that CD8^+^ T cell counts (AUC = 0.71, 95% CI: 0.571 - 0.853, *P* = 0.007; optimal cutoff: 233.65 cells/μL), CD4^+^ T cell counts (AUC = 0.70, 95% CI:0.570–0.847, *P* = 0.008; optimal cutoff: 630.49 cells/μL) (**Supplementary Figure 1**), and EGRIS (AUC = 0.80, 95% CI: 0.682–0.922, *P* < 0.001; optimal cutoff: 2.5) were associated with the need for mechanical ventilation in patients with GBS (**Figure 3A**). To evaluate the added value of T cell counts, we performed a combined ROC analysis integrating CD8^+^ T cell counts and CD4^+^ T cell counts with the EGRIS score. The combined model yielded an AUC of 0.87 (95% CI: 0.772–0.971, *P* < 0.001) (**Figure 3A**), representing an improvement over EGRIS alone (AUC = 0.80). These findings indicate that incorporating T cell counts with the EGRIS score enhances the accuracy of identifying GBS patients at high risk for requiring mechanical ventilation.

The present study demonstrated that the combined assessment of CD8^+^ T cell counts, CD4^+^ cell counts, and EGRIS score showed good discriminative ability for identifying patients with GBS who require mechanical ventilation. A decreased CD4^+^ T cell count in peripheral blood was associated with a higher likelihood of requiring mechanical ventilation. Further studies are needed to determine whether this phenomenon is associated with the migration of CD4^+^ T cells from peripheral blood into peripheral nerves, the extent of peripheral nerve inflammation and demyelination, and whether a marked reduction in circulating CD4^+^ cells reflects greater CD4^+^ T cell infiltration within peripheral nerves.

### 3.4 Accumulation of CD4^+^ T cells in the sciatic nerve of EAN rats

**Figure 4:**
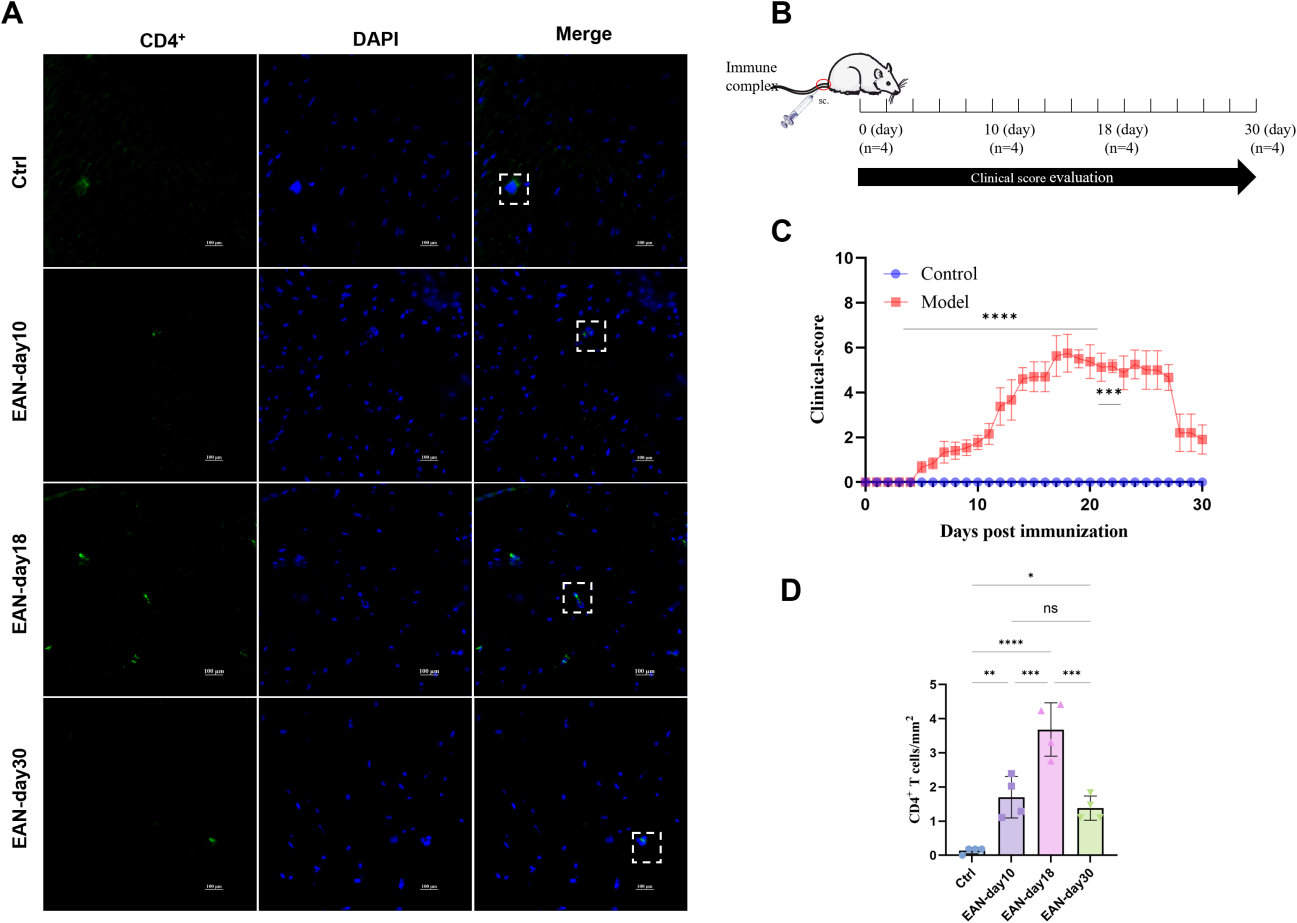
CD4^+^ T cell expression in the sciatic nerve of experimental autoimmune neuritis (EAN) rats, n = 4. **(A)** Immunofluorescence staining of rat sciatic nerve tissue. (original magnification, 40×). Green fluorescence indicates CD4^+^ T cells, blue fluorescence indicates DAPI, and the cells outlined in white and green represent CD4^+^ T cells. **(B)** Preparation of EAN rats, neurological function monitoring, and tissue collection timeline. **(C)** Neurological function score curve of EAN rats within 30 days after immunization. **(D)** Cell counts are expressed as mean ± standard deviation (SD). One-way ANOVA was used to assess the number of CD4^+^ T cells in the sciatic nerves of EAN rats at different disease stages, followed by Tukey’s *post hoc* test for multiple group comparisons. A p-value < 0.05 was considered statistically significant. Statistical differences between groups are indicated by asterisks: *P < 0.05; **P < 0.01; ***P < 0.001; ****P < 0.0001.

We established an EAN rat model of GBS and continuously monitored neurological function over a 30-day period (**Figures 4B, C**). Sciatic nerve samples were collected on days 10, 18, and 30 post-immunizations, representing the early, peak, and recovery phases of the disease, respectively. CD4^+^ T cells were labeled with green fluorescence using CD4^+^. In the EAN rat model, the number of CD4^+^ T cells in the sciatic nerve increased on day 10 post-immunization (1.70 ± 0.60 cells/mm^2^), peaked significantly on day 18 (3.68 ± 0.78 cells/mm^2^), and decreased markedly by day 30 (1.38 ± 0.35 cells/mm^2^), suggesting dynamic infiltration of CD4^+^ T cells during the disease course (**Figures 4A, D**). In contrast, patients with GBS had significantly lower numbers of peripheral blood CD4^+^ T cells than healthy controls.

## 4 Discussion

GBS generally follows a self-limiting clinical course; however, a subset of patients may develop severe complications, including respiratory failure requiring mechanical ventilation. Intravenous immunoglobulin (IVIG) and plasma exchange remain the primary therapeutic approaches for GBS. However, despite these treatments, approximately one-third of patients still require mechanical ventilation, 5% ultimately die, and about 20% experience poor long-term outcomes (10, 11). Súkeníková L and colleagues reported that patients with AIDP exhibited CD4^+^ T cells in their peripheral blood that responded to P2, P0, or PMP22 antigens (6). This study demonstrated that elevated CD8^+^ T cell counts and decreased NK cell counts in peripheral blood lymphocyte subsets were associated with an increased risk of GBS. Our results suggest that combining CD8^+^ T cell counts and CD4^+^ T cell counts with EGRIS scores shows enhanced discriminative ability in identifying GBS patients who required mechanical ventilation. This combined approach may help improve risk stratification in clinical practice; however, given the retrospective design of this study, further validation in prospective cohort studies is needed. Moreover, decreased peripheral blood CD4^+^ T cell counts were identified as an independent risk factor for mechanical ventilation, with lower CD4^+^ T cell levels corresponding to higher risk. In the EAN rat model, CD4^+^ T cell expression in the sciatic nerve reached peak levels. These results suggest that CD4^+^ T cells may migrate from the bloodstream into peripheral nerves, contributing to neuroinflammation and demyelination.

Previous studies have shown that patients with GBS exhibit a lower proportion of CD4^+^ T cells and a higher proportion of CD8^+^ T cells in peripheral blood compared with healthy individuals (12). In this study, the absolute counts of peripheral blood CD4^+^ and CD8^+^ T cells and the proportion of CD4^+^ cells were lower in GBS patients than in healthy controls, whereas the proportion of CD8^+^ T cells was comparable between the two groups. Notably, an increased absolute count of CD8^+^ T cells was independently associated with GBS. Yang M et al. reported that synergistic interactions between effector/memory CD8^+^ T cells and costimulatory macrophages are critical for initiating autoimmune-mediated peripheral neuropathy (13). Moreover, depletion of CD4^+^ T cells accelerate the onset of GBS and increases its likelihood, while CD8^+^ T cells may exert pathogenic effects during the development and progression of the disease (14).

Our study shows that both the absolute number and proportion of NK cells in the peripheral blood of patients with GBS are significantly reduced. Consistent with previous reports, NK cell activity decreases in peripheral blood after GBS onset, whereas it is markedly increased in the cerebrospinal fluid, suggesting a redistribution of NK cells to sites of inflammation. Moreover, the reported association between NK cell counts and the therapeutic efficacy of intravenous immunoglobulin in patients with chronic inflammatory demyelinating polyneuropathy (CIDP) further underscores the clinical relevance of NK cells in immune-mediated neuropathies (15–17). Together, these findings suggest that reduced NK cell levels may reflect impaired immune regulation, thereby increasing susceptibility to immune-mediated neurological injury.

In this study, lower NK cell counts were associated with a higher likelihood of developing GBS. In parallel, transcriptomic and bioinformatics analyses based on an *in vitro* myelination model using mouse dorsal root ganglion cultures revealed that NK cell–mediated cytotoxicity and B cell receptor signaling pathways may participate in peripheral myelination through molecules such as FCGR, RAC2, and PLCG2 (18). These observations indicate that NK cells may not only influence disease susceptibility but also contribute to the regulation of peripheral nerve myelin homeostasis.

A growing body of evidence indicates that NK cells play a crucial role in neuroimmune regulation and represent important potential therapeutic targets in neuroimmunology diseases (19). Reduced peripheral NK cell numbers or impaired cytotoxic function have been widely reported in several autoimmune disorders, including multiple sclerosis, rheumatoid arthritis, systemic lupus erythematosus, Sjögren’s syndrome, and type 1 diabetes mellitus. Meanwhile, the accumulation of NK cells in affected tissues—such as the pancreas in type 1 diabetes, hair follicles in alopecia areata, and muscle tissue in juvenile dermatomyositis—suggests that a decrease in circulating NK cells may reflect tissue recruitment rather than absolute depletion (20).

It should be noted, however, that although decreased peripheral NK cell levels were associated with increased susceptibility to GBS in our cohort, evidence from other inflammatory contexts indicates that NK cells may also exert pathogenic effects. For example, in patients with multiple sclerosis, viral infection can induce excessive NK cell activation, partly mediated by the activating receptor NKG2C, thereby promoting immune-mediated myelin injury (21). Taken together, these findings support the concept that NK cells play dual and context-dependent roles in immune-mediated neurological diseases: under physiological conditions, they may exert protective effects through immune regulation, whereas excessive or dysregulated activation in specific inflammatory environments may exacerbate tissue damage.

This study found that, compared with healthy controls, patients with GBS had a reduced absolute count of peripheral blood B cells but an increased relative proportion. Previous studies have similarly reported a marked elevation in peripheral blood B cell proportions during the acute phase of GBS, which correlates positively with Hughes scores. As the disease progresses, the proportion of B cells gradually declines, particularly during the early and late recovery phases (22). Brunn et al. found that B cells play a dual role in both the early and recovery phases of EAN, functioning as immunomodulators as well as pro-inflammatory mediators (23).

This study found that lymphocyte counts, NLR, and PLR in routine blood tests were associated with the need for mechanical ventilation in patients with GBS. Some studies have reported that routine blood NLR and PLR may serve as independent risk factors for severe GBS and the requirement for mechanical ventilation, and that their combination can provide effective predictive biomarkers (24, 25). Studies from multiple countries indicate that the modified Erasmus GBS respiratory failure score may serve as a practical clinical tool for predicting the risk of mechanical ventilation in patients with GBS (8, 26). This analysis included sex, age, CD8^+^ T cell count, CD4^+^ T cell count, and the EGRIS score. The results showed that older age, a higher EGRIS score, and a lower CD4^+^ T cell count were independently associated with the need for mechanical ventilation in patients with GBS. The Hughes and MRC scores were not included in the model because they were highly correlated with the EGRIS score.

Previous studies have shown that T cell-mediated immune responses against myelin proteins (especially P2 and P0) drive peripheral nerve injury (27, 28). In an EAN rat model, circulating T cells cross vascular endothelial cells and the basement membrane 10–15 days after immunization and adhere to the inner wall of small veins, leading to sciatic nerve demyelination (29). This study found that CD4^+^ T cell infiltration was increased in the sciatic nerve of EAN rats in the early, peak, and late stages of the disease, with the most significant increase in the peak stage. The reduction of peripheral blood CD4^+^ T cells in GBS patients may be related to the migration of CD4^+^ T cells from the blood circulation to the peripheral nerves (30). This study observed decreased peripheral blood CD4^+^ T cell counts in patients with GBS, along with increased CD4^+^ T cell infiltration in the sciatic nerves of EAN rats, which may indicate a potential role for CD4^+^ T cells in disease progression. However, direct comparisons between human and animal data should be interpreted with caution due to species differences and variations in sampling time points. Future studies are warranted to investigate changes in peripheral blood CD4^+^ T cells in EAN rats and their relationship with CD4^+^ T cell infiltration in the sciatic nerves, which could help clarify the potential link between systemic and local immune responses in GBS.

In conclusion, our study highlights the clinical relevance of peripheral blood lymphocyte subsets in GBS demonstrating their associations with disease severity and the need for mechanical ventilation. These findings underscore the potential value of immune profiling in risk stratification and clinical decision-making in patients with GBS.

Nonetheless, several limitations should be considered. The relatively small sample size limits the robustness of multivariable analyses, and validation in larger, independent cohorts is warranted. In addition, although immunofluorescence in the EAN rat model confirmed the presence of CD4^+^ T cells in peripheral nerves, this approach provided limited quantitative insight into immune cell infiltration. Moreover, direct evidence of CD4^+^ T cell involvement in human peripheral nerves remains lacking, and the target antigens in GBS have yet to be clearly defined. Therefore, the immune responses observed in this experimental model cannot be directly extrapolated to human disease.

Taken together, future studies integrating larger clinical cohorts with quantitative immune profiling in both experimental models and human tissues—including cerebrospinal fluid and, when feasible, peripheral nerve samples—will be essential to clarify the pathogenic role of specific lymphocyte subsets and to strengthen the translational relevance of these findings.

## Data Availability

The original contributions presented in the study are included in the article/**Supplementary Material**. Further inquiries can be directed to the corresponding authors.
