# Supplementary material for: Peripheral blood T-cell subsets combined with EGRIS score predict the need for mechanical ventilation in Guillain–Barré syndrome
Source: Front Immunol. 2026 Feb 4;17:1747416. doi: 10.3389/fimmu.2026.1747416 (PMC12913372; doi:10.3389/fimmu.2026.1747416)
Supplement: Supplementary file 1 [file DataSheet1.docx]

**Supplementary Data**

| Supplementary Table 1. Baseline characteristics of Guillain–Barré syndrome patients and healthy controls | | | | |
| --- | --- | --- | --- | --- |
|  | **HC**  **(n = 58)** | **GBS**  **(n = 55)** | ***χ2/Z*** | ***p*** |
| Gender |  |  | 3.879 | 0.049 |
| Male | 23 (39.7) | 32 (58.2) |  |  |
| Female | 35 (60.3) | 23 (41.8) |  |  |
| Age | 52.50 (45.75-58) | 56.00 (43.00-65.00) | 1.96 | 0.05 |

Note: Data are presented as median [interquartile range], or number (frequency percentage).

Abbreviations: HC, healthy controls; GBS, Guillain–Barré Syndrome.

| Supplementary Table 2. Blood Lipids and Peripheral Blood Inflammation-Related Indicators in Patients with GBS | | | | |
| --- | --- | --- | --- | --- |
|  | **No mechanical ventilation**  **(n = 26)** | **Mechanical ventilation**  **(n = 29)** | ***t/Z*** | ***p*** |
| CBC |  |  |  |  |
| Lymphocyte | 1.70 (1.39-3.04) | 1.00 (0.86-1.48) | 3.068 | 0.002 |
| NLR | 2.79 (1.60-3.61) | 9.15 (5.73-18.79) | 4.333 | < 0.001 |
| PLR | 102.98 (78.97-334.79) | 199.12 (116.97-302.42) | 2.293 | 0.022 |
| MLR | 0.44 (0.29-2.56) | 0.42 (0.26-0.65) | 0.716 | 0.474 |
| Blood lipids |  |  |  |  |
| Total cholesterol | 4.6252 ± 0.94095 | 3.9723 ± 1.04194 | 2.153 | 0.037 |
| TG | 1.60 (0.98-2.41) | 1.29 (0.99-1.81) | 1.154 | 0.248 |
| HDL | 0.9967 ± 0.44610 | 1.0977 ± 0.43125 | 0.755 | 0.454 |
| LDL | 2.7352 ± 0.65598 | 2.3468 ± 0.78203 | 1.76 | 0.086 |
| Apo A/Apo B | 1.04 (0.86-1.43) | 1.32 (1.01-1.59) | 1.1487 | 0.137 |

Note: Data are presented as mean ± standard deviation, median [interquartile range]. Lymphocyte count (10^9^/L). Blood lipids (mmol/L).

Abbreviations: CBC, complete blood count; NLR, neutrophil-to-lymphocyte ratio; PLR, platelet-to-lymphocyte ratio; MLR, monocyte-to-lymphocyte ratio; TG, triglyceride; HDL, high-density lipoprotein; LDL, low-density lipoprotein; Apo A, apolipoprotein A; Apo B, apolipoprotein B.


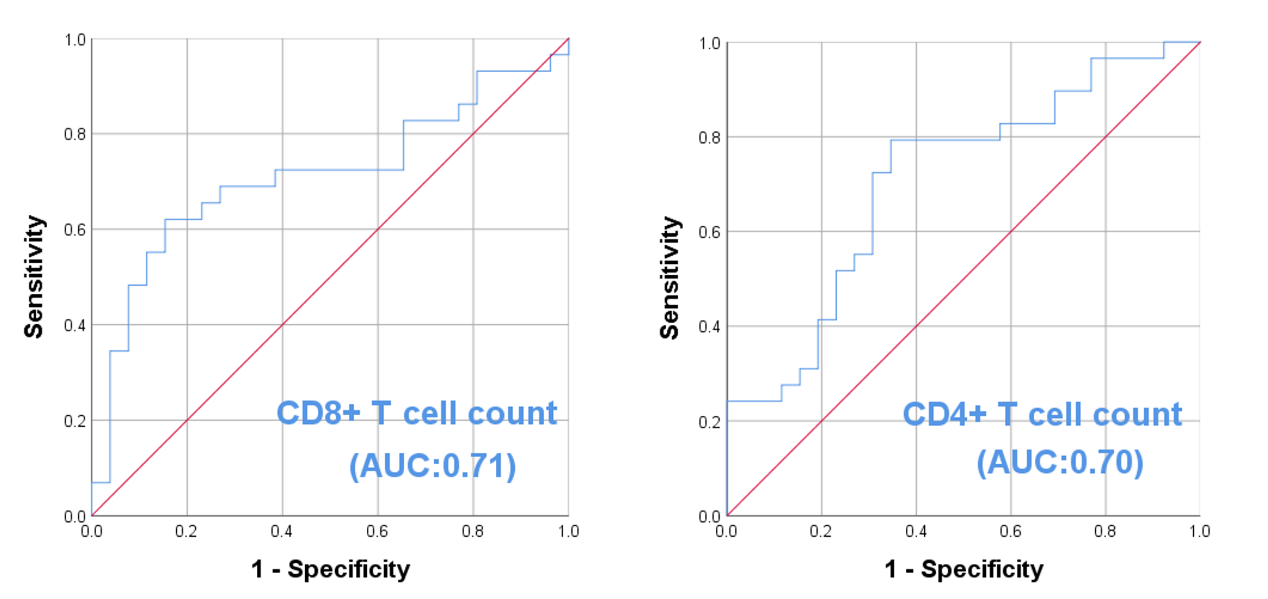


**Supplementary Figure 1.** Receiver operating characteristic (ROC) analysis of CD8⁺ and CD4⁺ T-cell counts for predicting the need for mechanical ventilation in patients with Guillain–Barré syndrome. A P value < 0.05 was considered statistically significant.
